# Langzeitverläufe bei Angststörungen

**DOI:** 10.1007/s00115-024-01789-0

**Published:** 2024-12-20

**Authors:** Jens Plag, Selina Heuer, Antonia Bendau, Andreas Ströhle

**Affiliations:** 1 Institute for Mental Health and Behavioral Medicine, Fakultät für Medizin,, HMU Health and Medical University Potsdam, Olympischer Weg 1, 14471 Potsdam, Deutschland; 2 Oberberg Fachklinik Potsdam, Potsdam, Deutschland; 3 Klinik für Psychiatrie und Psychotherapie, CCM, Charité – Universitätsmedizin Berlin, corporate member of Freie Universität Berlin and Humboldt Universität zu Berlin, Berlin, Deutschland

**Keywords:** Altersassoziierte Unterschiede, Symptomatik, Funktionsniveau, Therapie, Strukturelle Bildgebung, Age-associated differencess, Symptomatology, Functional level, Therapy, Structural imaging

## Abstract

**Hintergrund:**

Es existiert noch keine gemeinsame Übersicht über die langfristige Entwicklung des klinischen Bildes, erkrankungsassoziierter Einschränkungen sowie neurobiologischer Korrelate von Angststörungen und darüber, welchen Einfluss bestimmte Risikofaktoren sowie eine Behandlung auf die Prognose nehmen.

**Fragestellung:**

Dieser Beitrag stellt Befunde hinsichtlich der störungsspezifischen Symptomatik sowie erkrankungsassoziierter Veränderungen in den Bereichen des Funktionsniveaus, der Lebensqualität, der neurokognitiven Leistungen sowie der strukturellen Hirnanatomie über die Lebensspanne dar. Auch wird berichtet, wie personen- und umfeldbezogene Aspekte sowie eine leitliniengerechte Therapie den Erkrankungsverlauf beeinflussen.

**Material und Methoden:**

Zu den einzelnen Teilaspekten wurde eine Literatursuche in PubMed durchgeführt. Einbezogen wurden Metaanalysen, Longitudinal- und Kohortenstudien. Um Veränderungen über den Zeitverlauf zu illustrieren, erfolgte überwiegend eine getrennte Darstellung der Befunde für Kinder und Jugendliche sowie das frühe und späte Erwachsenenalter.

**Ergebnisse:**

Angststörungen weisen vor allem altersassoziierte Unterschiede im Symptomprofil, aber auch in den Bereichen Funktionsniveau und Lebensqualität auf. Insbesondere für das junge und mittlere Lebensalter konnten Risikofaktoren für einen ungünstigen Erkrankungsverlauf identifiziert werden. Befunde weisen jedoch darauf hin, dass eine evidenzbasierte Psycho- oder Pharmakotherapie auch nach ihrer Beendigung einen nachhaltigen Effekt besitzt.

**Diskussion:**

Für einen langfristige Therapieerfolg sollten altersabhängige Merkmale und Einschränkungen sowie prognostisch relevante Aspekte in der Diagnostik und Behandlung von Angststörungen beachtet und eine Behandlung zeitnah begonnen werden.

## Abstract (en)

**Background:**

At present there is no comprehensive overview of the long-term development of the clinical picture, disease-associated impairments and neurobiological correlates of anxiety disorders as well as what influence certain risk factors and treatment have on the prognosis.

**Objective:**

This article presents findings regarding disorder-specific symptoms and disorder-associated changes in the areas of functional level, quality of life, neurocognitive performance and structural brain anatomy over the lifespan. It also reports on how patient-related and environment-related aspects as well as guideline-oriented therapy influence the course of the disorder.

**Material and methods:**

A literature search was conducted in PubMed on the aspects of individual components. Meta-analyses, longitudinal and cohort studies were included. To illustrate changes over time, the findings were predominantly presented separately for children and adolescents as well as for early and late adulthood.

**Results:**

Anxiety disorders primarily exhibit age-associated differences in the symptom profile but also in the areas of functional level and quality of life. Risk factors for an unfavorable course of the disorder were identified particularly for young and middle-aged people; however, the findings indicate that evidence-based psychotherapy or pharmacotherapy also has a lasting effect even after termination.

**Conclusion:**

For long-term therapeutic success, age-dependent characteristics and limitations as well as prognostically relevant aspects in the diagnostics and treatment of anxiety disorders should be taken into consideration and treatment should be started as soon as possible.

Angststörungen sind bezüglich ihrer klinischen, epidemiologischen und pathogenetischen Charakteristika querschnittlich gut beschrieben. Wie sich erkrankungsassoziierte Befunde über die Lebensspanne entwickeln und wodurch Symptom- oder Therapieverläufe langfristig beeinflusst werden, ist jedoch deutlich weniger umfassend untersucht. Analog zu den weiteren Beiträgen dieser Ausgabe von *Der Nervenarzt* versuchen wir deshalb im Folgenden, Unterschiede im „Profil“ zwischen Altersgruppen herauszuarbeiten und darzustellen, wodurch die Krankheitsentwicklung beeinflusst wird und welche langfristigen Effekte von einer leitliniengerechten Behandlung zu erwarten sind.

## Methodik

**Abb. 1:**
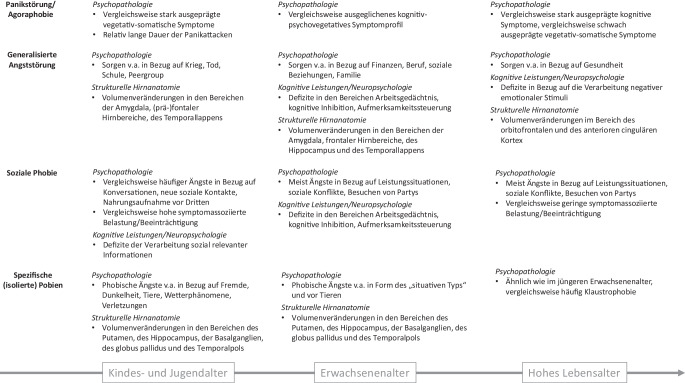
Übersicht über altersassoziierte Befunde bei Angststörungen

Die Literaturrecherche in PubMed wurde im Juni 2024 abgeschlossen. Im Rahmen der Suche wurden störungsspezifische Suchbegriffe mit alters- und domänenspezifischen Suchbegriffen kombiniert. Es erfolgte eine qualitative und quantitative Auswertung der Daten – d. h. wir betrachteten bei den einzelnen Angststörungen jeweils, ob sich die Art oder das Ausmaß der Veränderungen bzw. der Behandlungs- und Verlaufsprädiktoren in Abhängigkeit des Lebensalters verändert. In Bezug auf die jeweilige Fragestellung wurden zunächst Metaanalysen und gegebenenfalls einzelne Longitudinal- und Kohortenstudien in die Auswertung einbezogen. Neben der Darstellung im Text werden altersassoziierte Befunde zusätzlich als Übersicht durch Abb. 1 illustriert, sofern domänenspezifische Daten in Bezug auf mindestens zwei Altersgruppen ermittelt werden konnten. Es ist zu beachten, dass es sich bei der vorliegenden Arbeit um eine ausschnitthafte Darstellung der Befundlage und nicht um eine systematische Übersichtsarbeit handelt.

## Psychopathologie

### Agoraphobie und Panikstörung

Für die Agoraphobie und die Panikstörung liegen kaum Studien vor, die Besonderheiten der Psychopathologie in Abhängigkeit des Lebensalters explizit adressieren bzw. aus denen sich signifikante Unterschiede zwischen einzelnen Altersgruppen „herauslesen“ lassen. Dies liegt potenziell daran, dass die Erstmanifestationsgipfel beider Störungsbilder im mittleren Lebensalter verortet sind, das für sie typische Symptomprofil entsprechend überwiegend bei Personen zwischen dem 3. und 5. Lebensjahrzehnt sowie relativ homogen beschrieben wird und störungsspezifische Daten für das Kinder- und Jugendalter sowie das höhere Lebensalter kaum vorliegen. Dass dieser „Neglekt“ mindestens für die Agoraphobie, vor allem für den Bereich des Seniums ungerechtfertigt ist, legen epidemiologische Daten nahe, die auf die Relevanz bis in die Altersgruppe der über 60-Jährigen hindeuten [18].

In Bezug auf altersspezifische Symptomprofile bei der Panikstörung deuten Befunde bei *Kindern und Jugendlichen *darauf hin, dass im Vergleich zu Erwachsenen die vegetativ-somatischen Symptome stärker ausgeprägt sind als kognitive Phänomene, wie Kontrollverlusterleben oder Todesangst, und die durchschnittliche Dauer einer Panikattacke erhöht ist [10]. Im *Senium *scheint sich dieses Profil teilweise umzukehren. Beispielhaft lässt sich dies an einer Studie verdeutlichen, die über 300 Personen im Alter zwischen 60 und 95 Jahren mit Panikattacken einschloss und verschiedene Charakteristika der Symptomatik mit jüngeren Betroffenen verglich: Während sich Frequenz und subjektive Qualität der Panikattacken nicht zwischen den Altersgruppen unterschieden, waren kognitive Korrelate der Panik bei den Älteren stärker, die vegetativ-somatische Symptomatik jedoch schwächer ausgeprägt als bei den Jüngeren [8]. Diese Beobachtung korrespondiert mit den Befunden anderer Studien und wurde sowohl mit einer altersphysiologischen „Desensitivierung“ des vegetativen Nervensystems als auch mit einer altersassoziiert besseren Fertigkeit zur Affekt- bzw. Emotionskontrolle in Verbindung gebracht.

### Generalisierte Angststörung

Für die generalisierte Angststörung (GAS) ist bereits bekannt, dass sie sowohl bei Kindern als auch im mittleren und höheren Lebensalter eine hohe Prävalenz aufweist (z. B. [18, 28]). Vor diesem Hintergrund existieren zwar keine Longitudinalstudien, jedoch Querschnittsuntersuchungen, die sich insbesondere altersspezifischen Besonderheiten im Symptomprofil widmen.

Bei *Kindern *tritt die Erkrankung häufig zwischen dem 7. und 12. Lebensjahr auf und zeigt sich häufig in Form von Ängsten vor Krieg oder Tod, jedoch auch alltagsnah in Form von Sorgen, die sich auf die Leistungen in der Schule oder die Akzeptanz in der Peergroup beziehen. Im *mittleren Altersbereich*, der sich in Studien meist vom 3. bis ins 5. Lebensjahrzehnt erstreckt, liegen die Sorgenschwerpunkte inhaltlich meist in den Bereichen Finanzen, Beruf, soziale Beziehungen und Familie. Bei Betroffenen *ab dem 60. Lebensjahr* traten weiterhin primär familien- und ökonomiebezogene Sorgen auf – im Vergleich zu den Jüngeren zeigten sich jedoch zusätzlich signifikant häufiger Befürchtungen hinsichtlich des eigenen Gesundheitszustandes sowie der Gesundheit und Sicherheit von Angehörigen [11, 29].

Abseits der Sorgeninhalte konnten bisherige Untersuchungen keine altersabhängigen Unterschiede in Bezug auf verhaltensbezogene Phänomene der GAS (z. B. Rückversicherungs- oder Vermeidungsverhalten) oder das Ausmaß der symptomassoziierten psychosozialen Belastung feststellen. Wiederholt ergaben sich jedoch Hinweise darauf, dass die „Sorgenfrequenz“, d. h. die Auftretenshäufigkeit der störungsspezifischen Symptomatik, mit steigendem Lebensalter signifikant abnimmt (z. B. [11]) – ein Befund, der u. a. mit wechselnden Alltagsanforderungen, Habituationsprozessen oder einer verbesserten Emotionsregulation erklärt wird.

### Soziale Phobie

Die soziale Phobie zeigt eine relativ hohe und ansteigende Prävalenz im Kindes- und Jugendalter, die im mittleren und höheren Alter hingegen kontinuierlich abnimmt [22]. Die Art der sozialphobischen Trigger kann interindividuell sehr unterschiedlich sein und die Symptomatik nur auf einen bestimmten Kontext bezogen auftreten (z. B. als „Performance-only-Subtyp“ in Situationen, in der die Betroffenen eine eigene Leistung präsentieren müssen) oder allgemein durch die soziale Bewertung durch Dritte ausgelöst werden. Auch bezüglich der sozialen Phobie existieren unseres Wissens bisher keine Längsschnittuntersuchungen, die einen möglichen „Shift“ des Symptomprofils untersucht haben. Untersuchungen älteren Datums weisen jedoch mittel- bis langfristig darauf hin, dass sich subklinische soziale Ängste inhaltlich kaum zwischen jüngeren und älteren Menschen unterscheiden (z. B. [6]).

Im Jahr 2015 wurde eine Studie mit über 80 Personen mit einer sozialen Phobie durchgeführt, um mögliche altersassoziierte Unterschiede zu detektieren [29]. Vor diesem Hintergrund wurden zwei Gruppen gebildet, die die Altersbereiche zwischen 18 und 44 Jahren sowie zwischen 60 und 84 Jahren abbildeten. Hier zeigte sich ebenfalls, dass beide Gruppen einen hohen Überschneidungsbereich in Bezug auf die erkrankungsspezifischen Auslösesituationen aufwiesen: Die Angst vor verbaler Beteiligung vor Publikum, selbstbewusstem Auftreten in Konfliktsituationen, dem Zurückweisen von Anliegen anderer sowie das Besuchen von Partys waren „transgenerational“ relevant. Signifikant häufiger waren bei *Jüngeren* jedoch zusätzlich Ängste vorhanden, die sich auf Konversationen, den Kontakt mit unbekannten Menschen sowie die Nahrungsaufnahme in der Öffentlichkeit bezogen. Dies korrespondierte mit einer signifikant höheren symptombezogenen Belastung und Beeinträchtigung in dieser Altersgruppe verglichen mit den Älteren [29].

Die Beobachtung, dass *ältere Menschen* weniger Leidensdruck und Einschränkungen im Kontext sozialer Ängste erleben, teilen auch andere Studien [6, 14]. Dies könnte dadurch begründet sein, dass ältere Menschen mehr Erfahrung im Umgang mit gefürchteten Situationen aufweisen oder einige sozialphobische Auslösesituationen mit steigendem Lebensalter zunehmend weniger alltagsrelevant sind und so besser vermieden werden können.

### Spezifische (isolierte) Phobien

Die spezifischen Phobien bilden sich in allen Altersbereichen mit einer vergleichsweise hohen Prävalenz von bis zu 12 % ab [18]. Die in der International Statistical Classification of Diseases and Related Health Problems 10 (ICD-10) und in Abhängigkeit des phobisch besetzen Kontexts definierten Subkategorien des „Tier-“, „Umwelt-“ und „Blut-Injektions-Verletzungs-Typs“ sowie des „situativen Typs“ besitzen jedoch insbesondere im Vergleich zwischen den verschiedenen Altersbereichen eine unterschiedliche Relevanz.

Vor dem Hintergrund der Tatsache, dass phobisches Erleben und Verhalten in bestimmten kindlichen Entwicklungsstufen durchaus als normalpsychologisches „Durchgangsphänomen“ interpretiert werden kann, legen die Befunde von Kohortenstudien nahe, dass *Kleinkinder* eine phobische Reaktion insbesondere gegenüber fremden Menschen, Dunkelheit und Tieren entwickeln. Diese Auslöser werden etwa ab dem 6. Lebensjahr schließlich um Ängste mit Bezug auf Wetterphänomene und potenzielle körperliche Verletzung ergänzt. Im *Jugendalter* scheinen eher Blut und Verletzungen sowie Naturkatastrophen als Trigger zu dominieren; gleichzeitig lässt sich ab diesem Alter bereits eine Entwicklung eines unterschiedlichen Situations- und Objektbezugs feststellen [25]. Für das *junge und mittlere Erwachsenenalter* konnte gezeigt werden, dass unterschiedliche Varianten des „situativen Typs“ – häufig in Form einer Höhenphobie, Klaustrophobie oder Furcht vor (zahn)ärztlicher Diagnostik/Behandlung – deutlich dominieren, gefolgt vom „Umwelt-“ und „Tier-Typ“.

Wie auch bei den anderen Angsterkrankungen ist die Datenlage für das höhere Alter auch bei der spezifischen Phobie eingeschränkt. Zwei Studien untersuchten die Profile von Personen mit einer spezifischen Phobie, die *älter als 60 Jahre* waren, und verglichen diese (un)mittelbar mit einer Gruppe jüngerer Erwachsener [13, 29]. Durch beide Arbeiten wurde prinzipiell das gleiche Muster der Subtypen wie bei jüngeren Erwachsenen gefunden („situativer Typ“ vor „Umwelt-“ und „Tier-Typ“), wobei sich jedoch zumindest Hinweise darauf ergaben, dass eine Klaustrophobie bei den Älteren vergleichsweise häufig vorkommt. Ähnlich wie bei der GAS fanden sich keine altersassoziierten Unterschiede in Bezug auf die psychosoziale Belastung und Beeinträchtigung, die aus der Erkrankung resultieren [29].

## Psychosoziales Funktionsniveau und Lebensqualität

Es erscheint intuitiv, dass Angststörungen in allen Lebensabschnitten mit ausgeprägten Einschränkungen des psychosozialen Funktionsniveaus und der Lebensqualität einhergehen, neben der unmittelbaren symptomassoziierten psychischen Belastung nicht zuletzt durch das erkrankungsimmanente Sicherheits- und Vermeidungsverhalten bedingt. Eine relativ große Anzahl an Querschnittsuntersuchungen konnte zeigen, dass Angststörungen – unabhängig von ihrem Subtyp – bereits bei *Kindern und Jugendlichen* zu einer signifikanten Beeinträchtigung in unterschiedlichen Lebensbereichen führen. Hierzu zählen der familiäre Kontext (z. B. Schwierigkeiten bei der Teilnahme an Familienaktivitäten oder -routinen), die schulische Situation (v. a. schlechtere schulische Leistungen u. a. durch häufiges erkrankungsbedingtes Fehlen) sowie gestörte Beziehungen zu Gleichaltrigen, die mit größerer Einsamkeit und einem Verlust an sozialer Kompetenz verbunden sind (z. B. [7]). Darüber hinaus konstatierten einige Kohortenstudien in dieser Altersgruppe im Vergleich zu Menschen ohne Angststörungen signifikante Einschränkungen in unterschiedlichen Domänen der Lebensqualität wie dem körperlichen und psychischen Wohlbefinden, der autonomen Lebensführung und der Qualität der Beziehung zu Eltern- und Geschwistern [27].

Im Jahr 2024 wurde eine Metaanalyse publiziert, die negative Effekte von Angststörungen auf das psychosoziale Funktionsniveau in der Altersgruppe von 7 bis 17 Jahren quantifizierte. Unter Einschluss von insgesamt 9 vergleichsweise hochwertigen Studien wurde hier insgesamt ein großer statistischer Effekt gefunden [9]. Zusammen mit ebenfalls großen Effekten, die sich für die Minderung der Lebensqualität aus den entsprechenden Einzelstudien ergeben, unterstreicht dieser Befund noch einmal den stark invalidisierenden Einfluss, der aus einer Angststörung bereits für junge Menschen resultiert.

Bei *Erwachsenen* vermittelt nach wie vor eine Metaanalyse aus dem Jahr 2007 den umfangreichsten Eindruck in Bezug auf die mit einer Angststörung assoziierten Veränderungen des psychosozialen Funktionsniveau bzw. der Lebensqualität [19]. Diese umfasst 23 kontrollierte Studien, die die Panikstörung, die GAS oder die soziale Phobie adressierten und Personen einschlossen, die im Mittel 39 Jahre alt waren. Neben einer signifikanten Reduktion der allgemeinen Lebensqualität im Vergleich zu Gesunden üben alle Angststörungen statistisch jeweils große negative Effekte auf das private und berufliche Funktionsniveau sowie auf die gesundheitsbezogene Lebensqualität aus – sowohl im somatischen als auch im psychischen Bereich. Der Vergleich der einzelnen Angststörungen deutet darauf hin, dass insbesondere das psychische Wohlbefinden bei Personen, die von einer GAS oder einer Panikstörung betroffen sind, stärker beeinträchtigt ist als bei denjenigen, die an einer sozialen Phobie leiden – ein Befund, der gut zu der bereits weiter oben berichteten negativen Assoziation zwischen der symptomassoziierten Belastung sowie Beeinträchtigung und dem Lebensalter passt.

Untersuchungen, welche die Lebensqualität bei Angststörungen im *Senium *zum Gegenstand haben, existieren kaum. Eine kontrollierte Studie, in der 164 Personen mit einer GAS ab einem Alter ab 65 Jahren eingeschlossen wurden, konnte ebenfalls eine signifikant ausgeprägtere Minderung der gesundheitsbezogenen Lebensqualität durch die Angststörung detektieren [21] und zumindest einen Hinweis darauf liefern, dass dieser Aspekt über die Lebensspanne relevant bleibt.

## Kognitive Leistungen/Neuropsychologie

Vergleichbar zu anderen in diesem Beitrag behandelten Themen liegt in Bezug auf Angststörungen für den Bereich der kognitiven bzw. neuropsychologischen Leistungen bisher ebenfalls keine Longitudinaluntersuchung vor, die einen Eindruck darüber vermittelt, wie sich bestimmte Domänen in einer stabilen Gruppe betroffener Personen über einen definierten Zeitraum entwickeln. Mit Ausnahme der spezifischen Phobien existieren jedoch für alle in der ICD-10 gelisteten Subtypen insbesondere vom jungen bis zum höheren Erwachsenenalter Querschnittsuntersuchungen bzw. diese zusammenfassende Übersichtsarbeiten, die diesen Bereich zum Gegenstand haben.

Während für den Altersbereich der *Kinder und Jugendlichen* Daten zur Panikstörung und GAS bisher fehlen, konnte für die soziale Phobie bereits eine relativ große Anzahl an Befunden in dieser Altersgruppe erhoben werden, die mit der Fähigkeit zur Verarbeitung sozial relevanter Informationen zusammenhängen. Im Vergleich zu Gesunden zeigten Erkrankte im Rahmen standardisierter Testreihen wiederholt vor allem eine Wahrnehmungsverzerrung hin zu bedrohlichen sozialen Stimuli sowie eine signifikant erhöhte Tendenz, mit Unsicherheit besetzte Situationen als bedrohlich wahrzunehmen sowie die eigene Leistung im Kontext sozialer Bewertung zu unterschätzen [15]. Es scheint naheliegend, dass diese Befunde zentral mit der Entstehung und Aufrechterhaltung sozialer Ängste verknüpft sind.

Für den *jungen und mittleren Erwachsenbereich* liegen vor allem Ergebnisse vor, die bei der Panikstörung, der GAS und der sozialen Phobie auf Beeinträchtigungen impliziter bzw. expliziter Gedächtnisleistungen, des Arbeitsgedächtnisses, der kognitiven Inhibition sowie der Aufmerksamkeitssteuerung hinweisen (z. B. [5]). Insbesondere die beiden letztgenannten Faktoren gelten mittlerweile als zentrale und im Kontext von Angststörungen transdiagnostisch relevante Pathogenesefaktoren.

Auch wenn für das *höhere Lebensalter* wenige Arbeiten vorliegen, konnten einige der genannten Befunde auch in diesem Lebensabschnitt bei allen genannten Angststörungen repliziert werden. Interessant erscheinen zudem die Ergebnisse einer relativ aktuellen Übersichtsarbeit, die Daten in Bezug auf die Emotionsverarbeitung bei Menschen mit einer GAS *ab einem Alter von 66 Jahren* zusammengetragen hat und Hinweise dafür fand, dass diese im Vergleich zu Nichtbetroffenen eine Beeinträchtigung in der Verarbeitung negativer emotionaler Stimuli aufweisen [12].

## Einfluss von Behandlung auf den Langzeitverlauf

Angststörungen weisen unbehandelt meist einen chronischen bzw. durch wiederholte Rezidive geprägten Verlauf auf [24]. Entsprechend ist es einigermaßen von Bedeutung, die Effekte von v. a. erstrangigen Behandlungsverfahren wie Psycho- und Pharmakotherapie auf die langfristige Symptomentwicklung abschätzen und bewerten zu können.

Obwohl sich insbesondere neuere Antidepressiva auch bei Angststörungen im *Kindes- und Jugendalter* als wirksam erwiesen haben, liegen in dieser Altersgruppe bisher keine Daten in Bezug auf ihre Effekte auf den Langzeitverlauf vor. In Bezug auf die Beurteilung der nachhaltigen Wirksamkeit einer Psychotherapie bei jungen Betroffenen hat die Metaanalyse von Barry und Kolleg:innen in der jüngeren Vergangenheit einen relativ großen Beitrag geleistet [4]. Unter Einschluss von mehr als 140 randomisiert-kontrollierten Studien (RCT) wurde berechnet, ob sich die Effekte einer expositionsbasierten kognitiven Verhaltenstherapie (KVT) bei Personen mit einer Panikstörung, einer GAS, einer sozialen oder einer spezifischen Phobie in Abhängigkeit ihres Alters voneinander unterscheiden. Hierzu wurden zwei Altersgruppen gebildet (11 bis 18 Jahre vs. 19 bis 59 Jahre) und jeweils die Behandlungseffekte unmittelbar nach Abschluss der KVT sowie zu einem ggf. durchgeführten Katamnesezeitpunkt ermittelt. Es zeigte sich, dass die Gruppe der Älteren unmittelbar nach Abschluss der Behandlung eine ausgeprägtere Symptomreduktion aufwies und diese langfristig relativ stabil blieb. Die Jüngeren zeigten jedoch innerhalb des Nachbeobachtungszeitraumes eine weitere deutliche Symptomverbesserung, sodass zwischen beiden Gruppen zur Katamnese kein Unterschied mehr bestand. Überlegungen zu dieser Beobachtung waren dahingehend, dass die der KVT zugrunde liegenden Lernmechanismen bzw. deren Konsolidierung im jüngeren Alter möglicherweise mehr Zeit in Anspruch nehmen oder erst nach längerer „Beübung“ alltagsrelevant werden.

Auch für das *mittlere Erwachsenenalters *existiert zu diesem Thema eine aussagekräftige Metaanalyse [3]. In dieser wurden die Ergebnisse von insgesamt 94 RCT ausgewertet, welche die Effekte unterschiedlicher psycho-, jedoch auch pharmakotherapeutischer Behandlungsstrategien bei der Panikstörung, der GAS oder der sozialen Phobie untersuchten sowie zusätzlich jeweils eine Katamneseuntersuchung mit einem zeitlichen Abstand zum Therapieende von bis zu 2 Jahren durchführten. Die in den eingeschlossenen Studien am häufigsten verwendete psychotherapeutische Technik war die KVT; die weiteren Methoden umfassten psychodynamische, interpersonelle, achtsamkeitsbasiere und auf Entspannung ausgerichtete Therapieansätze, welche für die metaanalytische Auswertung als „andere Psychotherapien“ zusammengefasst wurden. Die dritte Gruppe der Medikamente beinhaltete neuere Substanzen aus den Klassen der selektiven Serotoninwiederaufnahmehemmer und der Monoaminooxidasehemmer, das Trizyklikum Imipramin sowie verschiedene Benzodiazepine. Durch die metaanalytische Auswertung konnte transdiagnostisch gezeigt werden, dass die durch alle drei aktiven Therapiemaßnahmen erreichte signifikante Symptomverbesserung bis zur Katamnese mindestens aufrechterhalten wurde. Ausschließlich durch die KVT konnte jedoch zusätzlich eine weitere Reduktion der erkrankungsspezifischen Symptomatik zwischen dem Ende der Behandlung und dem Nachbeobachtungszeitpunkt erzielt werden [3].

Obwohl die Wirksamkeit einer leitliniengerechten Psycho- und Pharmakotherapie auch bei Angststörungen im *Senium* empirisch abgesichert ist, liegen bis heute keine Befunde vor, die Schlussfolgerungen dahingehend zulassen, ob auch in dieser Altersgruppe langfristige Behandlungseffekte vorhanden sind. In der Zusammenschau deutet die Datenlage jedoch darauf hin, dass die gegenwärtigen leitliniengerechten Therapieoptionen – und hier insbesondere die KVT – auch nach ihrer Beendigung zu einem positiven Verlauf von Angststörungen beitragen.

## Faktoren für die Langzeitprognose

In den vergangenen Jahren wurden häufig psychologische, demographische, psychosoziale, biographische sowie geschlechtsspezifische Aspekte intensiv dahingehend untersucht, ob sie den Verlauf bzw. die Prognose von Angststörungen beeinflussen. Die verfügbaren Befunde der bisherigen Kohorten- und teils auch Longitudinaluntersuchungen ergeben diesbezüglich nicht immer ein eindeutiges Bild.

Untersuchungen im *Jugend-* bis hin zum *Erwachsenenalter *weisen auf mehrere Faktoren hin, die häufig mit einer langfristig höheren Symptomausprägung über die Zeit sowie einem größeren Risiko für einen chronischen Verlauf bzw. häufige Symptomrezidive verbunden sind. Neben störungsbezogenen Aspekten (z. B. längere Dauer, höherer Schweregrad, ein länger bestehendes Vermeidungsverhalten, stärkere Beeinträchtigungen, eine höhere Anzahl an Panikattacken), dem Vorliegen komorbider psychischer (v. a. Persönlichkeitsstörungen) oder somatischer Erkrankungen und dem weiblichen Geschlecht scheinen weitere umwelt- und entwicklungsbedingte Risikofaktoren vorzuliegen, wie ein geringes Geburtsgewicht, elterlicher Substanzgebrauch oder frühe Missbrauchserfahrungen. Die Ergebnisse verdeutlichen darüber hinaus den negativen Einfluss demographischer Risikofaktoren auf den Erkrankungsverlauf (z. B. niedriger sozioökonomischer Status und Bildungsgrad, alleinstehend und kinderlos). Während der *Adoleszenz* sowie im *mittleren Erwachsenenalter* scheinen Persönlichkeitsmerkmale wie erhöhte Werte in Bezug auf Neurotizismus, Angstsensitivität und Verhaltenshemmung ein niedriger Selbstwert mit erhöhten Rezidivraten assoziiert zu sein [2, 17, 26].

## Strukturelle Bildgebung

Schwerpunktmäßig seit Anfang der 2000er-Jahre wurden kontrollierte Studien mittels Magnetresonanztomographie durchführt, die die strukturelle Hirnanatomie von Angststörungen abbilden. Ein Fokus lag hierbei auf den Bereichen des sog. „Angst-“ oder „Furchtnetzwerks“, zu dem die Amygdala, der Hippokampus, der Hypothalamus, die Insula, präfrontale Hirnbereiche und weitere (para-) limbische Strukturen gehören und welches für die Wahrnehmung und Bewertung potenziell bedrohlicher Stimuli sowie für die Auslösung der psychophysiologischen Angstreaktion von zentraler Bedeutung ist. Während Longitudinalstudien auch zu diesem Thema bis heute fehlen, liegen mittlerweile für alle in der ICD-10 gelisteten Angststörungen einige Querschnittsuntersuchungen und Metaanalysen vor. Insbesondere für die GAS wird durch diese ein relativ großes Altersspektrum abgedeckt. Obwohl vielfach eine erkrankungsassoziierte Volumenveränderung einzelner Hirnstrukturen sowie eine Korrelation dieser Befunde mit der Symptomschwere gefunden wurde, zeichnet sich die Datenlage in jedem Lebensalter sowie für die einzelnen Subtypen durch eine große Heterogenität aus. Dies lässt vermuten, dass das Erkrankungsrisiko in diesem Bereich am ehesten auf eine Störung der strukturellen bzw. funktionellen „Homöostase“ und nicht auf einzelne Veränderungen zurückführbar ist.

### Panikstörung mit/ohne Agoraphobie

Kontrollierte Studien, die Kinder- und Jugendliche sowie Menschen im Senium zum Gegenstand haben, liegen bisher nicht vor. Im Vergleich zu Menschen ohne Angststörungen wurden für betroffene *Erwachsene im mittleren Lebensalter* wiederholt insbesondere Volumenminderungen in den Bereichen der Amygdala, der Insula, verschiedener Bereiche des Vorderhirns wie beispielsweise dem orbitofrontalen (OFC) und anterioren cingulären Kortex (ACC) sowie im Temporallappenbereich gefunden. Durch eine aktuelle Metaanalyse wurde für diese Altersgruppe ausgewertet, ob sich die Befunde in Abhängigkeit eines „frühen“ und eines „späten“ Erstmanifestationszeitpunktes der Erkrankung unterscheiden: Ein früher Beginn war mit einer signifikanten Verkleinerung der Amygdala und des linken ACC assoziiert, ein spätes Auftreten hingegen mit einer Größenabnahme von Hirnarealen, die mit der Insula strukturell zusammenhängen [20].

### GAS

Für die GAS existieren Befunde für alle Altersgruppen. Bei *Kindern und Jugendlichen* wurde in jeweils ungefähr gleichem Maß sowohl eine Volumenminderung als auch -vergrößerung in den Bereichen der Amygdala und (prä-)frontaler Hirnbereiche objektiviert; wiederholt fand sich im Vergleich zu Menschen ohne Angststörungen eine Volumenzunahme im Bereich des Temporallappens. Letzteres konnte auch für das *junge und mittlere Erwachsenalter* dargestellt werden. In diesem Lebensabschnitt zeigte sich zusätzlich die Tendenz hin zu einer Verkleinerung frontaler Areale sowie des Hippokampus, aber auch zu einer Vergrößerung der Amygdala (z. B. [23]). Bei Betroffenen im *Senium* fanden sich schließlich primär Veränderungen des OFC und des ACC, jedoch durchaus in unterschiedliche Richtungen (z. B. [1]).

### Soziale Phobie

Bei der sozialen Phobie fokussierten die bisherigen Arbeiten auf Personen unter 15 Jahren und Befunde zum höheren Erwachsenenalter und zum Senium fehlen. Die Studien, die bei *Kindern, Jugendlichen* sowie *jungen Erwachsenen *durchgeführt wurden, berichten – vergleichbar mit anderen Angststörungen – von Veränderungen der Amygdala, des OFC und weiterer präfrontaler Areale, der Insula, aber auch des Hippokampus und des Thalamus (z. B. [30]). In der Zusammenschau gehen diese jedoch in unterschiedliche Richtungen, ohne dass ein klares „Muster“ zu erkennen ist.

### Spezifische Phobien

Unter Einschluss von mehr als 4000 Personen untersuchte die erst kürzlich publizierte und umfangreichste Metaanalyse störungsspezifische hirnstrukturelle Veränderungen spezifischer Phobien [16]. In die Auswertung wurden alle phobischen Subtypen sowie Betroffene im Alter von 9 bis 51 Jahren eingeschlossen. Im Vergleich zu Personen ohne Angststörungen war das Vorliegen einer spezifischen Phobie mit reduzierten Hirnvolumina in den Bereichen des Putamen und Hippokampus, jedoch auch innerhalb der Basalganglien verbunden. Signifikante Volumenvergrößerungen wurden v. a. für den Globus pallidus und den Temporalpol beschrieben. In den Subanalysen zeigte sich, dass in Bezug auf diese Befunde weder zwischen den einzelnen Arten spezifischer Phobien noch zwischen *Kinder und Jugendlichen* (< 21 Jahren) und *Erwachsenen* signifikante Unterschiede bestanden. Daten, die sich auf Betroffene im Senium beziehen, liegen bisher nicht vor.

## Fazit für die Praxis

- In Abhängigkeit des Lebensalters können Menschen mit Angststörungen Unterschiede im Symptomprofil, aber auch hinsichtlich ihres Funktionsniveaus, der neurokognitiven Leistungen und ihrer Lebensqualität aufweisen.
- Gemeinsam mit Risikofaktoren für einen ungünstigen Erkrankungsverlauf, die bereits im jungen Lebensalter identifizierbar sind, sollten diese beachtet werden, um den nachhaltigen Effekt leitlinienbasierter Psycho- oder Pharmakotherapie bestmöglich nutzbar zu machen.
